# Treatment envelope evaluation in transcranial magnetic resonance-guided focused ultrasound utilizing 3D MR thermometry

**DOI:** 10.1186/2050-5736-2-19

**Published:** 2014-10-16

**Authors:** Henrik Odéen, Joshua de Bever, Scott Almquist, Alexis Farrer, Nick Todd, Allison Payne, John W Snell, Douglas A Christensen, Dennis L Parker

**Affiliations:** 1 Utah Center for Advanced Imaging Research, Department of Radiology, University of Utah, Salt Lake City, Utah 84108, USA; 2 Department of Physics and Astronomy, University of Utah, Salt Lake City, Utah 84112, USA; 3 School of Computing, University of Utah, Salt Lake City, Utah 84112, USA; 4 Department of Bioengineering, University of Utah, Salt Lake City, Utah 84112, USA; 5 Focused Ultrasound Foundation, Charlottesville, Virginia 22903, USA; 6 Department of Neurological Surgery, University of Virginia, Charlottesville, Virginia 22908, USA; 7 Department of Electrical and Computer Engineering, University of Utah, Salt Lake City, Utah 84112, USA

**Keywords:** Treatment envelope, tcMRgFUS, MR thermometry, PRF, Brain

## Abstract

**Background:**

Current clinical targets for transcranial magnetic resonance-guided focused ultrasound (tcMRgFUS) are all located close to the geometric center of the skull convexity, which minimizes challenges related to focusing the ultrasound through the skull bone. Non-central targets will have to be reached to treat a wider variety of neurological disorders and solid tumors. Treatment envelope studies utilizing two-dimensional (2D) magnetic resonance (MR) thermometry have previously been performed to determine the regions in which therapeutic levels of FUS can currently be delivered. Since 2D MR thermometry was used, very limited information about unintended heating in near-field tissue/bone interfaces could be deduced.

**Methods:**

In this paper, we present a proof-of-concept treatment envelope study with three-dimensional (3D) MR thermometry monitoring of FUS heatings performed in a phantom and a lamb model. While the moderate-sized transducer used was not designed for transcranial geometries, the 3D temperature maps enable monitoring of the entire sonication field of view, including both the focal spot and near-field tissue/bone interfaces, for full characterization of all heating that may occur. 3D MR thermometry is achieved by a combination of *k*-space subsampling and a previously described temporally constrained reconstruction method.

**Results:**

We present two different types of treatment envelopes. The first is based only on the focal spot heating—the type that can be derived from 2D MR thermometry. The second type is based on the relative near-field heating and is calculated as the ratio between the focal spot heating and the near-field heating. This utilizes the full 3D MR thermometry data achieved in this study.

**Conclusions:**

It is shown that 3D MR thermometry can be used to improve the safety assessment in treatment envelope evaluations. Using a non-optimal transducer, it is shown that some regions where therapeutic levels of FUS can be delivered, as suggested by the first type of envelope, are not necessarily safely treated due to the amount of unintended near-field heating occurring. The results presented in this study highlight the need for 3D MR thermometry in tcMRgFUS.

## Background

Focused ultrasound (FUS) is a promising treatment modality for a wide range of disorders due to its precise, non-invasive, and ionizing radiation-free nature. It is currently being performed under ultrasound [1-4] (US) and magnetic resonance (MR) guidance [5-7]. Both thermal and mechanical [8-11] effects of the US interaction with tissues are being utilized, and current targets of clinical interest include the prostate [12-14], liver [15-17], breast [18-20], uterine fibroid [21,22], and brain [5,23-25].

Neurological applications of FUS have been of particular research interest due to the unique possibility of non-invasive delivery of US energy through the intact skull bone to a precise location within the brain [26-28]. Irreversible surgical procedures utilizing high US intensities for tissue ablation [24,29,30] as well as reversible neuro-modulation and blood-brain barrier disruption procedures utilizing lower US intensities [31-35] have been investigated. Challenges to transcranial FUS have included the defocusing of the US beam due to phase aberrations by the skull bone [36], and the difficulty of non-invasively monitoring the procedures. Recent approaches to phase aberration correction [37-43] have greatly improved the ability to maintain a localized US focal spot even after propagation through the skull. Non-invasive treatment monitoring has been made possible with the use of magnetic resonance imaging (MRI). MRI offers the ability to acquire high-resolution anatomical images with various contrasts in arbitrary imaging planes, real-time feedback from MR thermometry, as well as methods for post-treatment evaluation [44-46]. For MR temperature imaging (MRTI), various temperature-sensitive MR parameters such as the T1 and T2 relaxation times can be utilized [47,48]; however, the current gold standard for temperature measurements in aqueous tissues is the proton resonance frequency (PRF) shift method [49,50]. The primary challenge in MR thermometry for transcranial applications is achieving accurate and precise temperature measurements with adequate spatial and temporal resolution over a sufficiently large field of view (FOV). Typical MRTI methods cover only one or a few two-dimensional (2D) slices in order to get high enough spatio-temporal resolution for accurate temperature measurements [23,24].

Despite recent improvements in phase aberration correction and non-invasive treatment monitoring, current clinical targets in transcranial MR-guided FUS (tcMRgFUS) are all close to the geometric center of the skull convexity [23,24]. For current clinical tcMRgFUS systems, which utilize large hemispherical phased-array US transducers, this minimizes the challenges of focusing through the skull, as most of the ultrasound elements will be nearly orthogonal to the skull surface. Central focusing also results in less unintended off-focal heating (i.e., heating in near- and far-field tissue/bone interfaces, and heating due to grating lobes) than may occur when focusing close to the skull. However, to be able to treat a wider variety of neurological disorders and solid tumors, therapeutic levels of FUS will have to be delivered to non-central targets within the skull as well. This will lead to more challenging beam aberrations and more unintended off-focal heating. To investigate in what regions of the brain therapeutic levels of FUS can be delivered, Eames et al. [51] and Dallapiazza et al. [52] performed treatment envelope studies where the US focus was mechanically and electronically moved to various locations within human cadaver heads and phantom skulls while monitoring the heating with 2D MRTI.

In this work, we present methods to perform treatment envelope studies utilizing three-dimensional (3D) MRTI by combining *k*-space subsampling and a previously described constrained reconstruction method. The utilization of 3D MRTI enables monitoring of the fully insonified FOV and thereby simultaneous monitoring of the focal spot and any unintended near-field heating. The methodology of this proof-of-concept study is evaluated in both a phantom and a lamb model, and it is shown that although therapeutic levels of US can be delivered to an intracranial region, the amount of unintended heating occurring in the near-field may make it unsafe to do so.

## Methods

All MR imaging was performed with a 3D gradient recalled echo pulse sequence with segmented echo planar imaging readout on a 3 T MRI scanner (TIM Trio, Siemens Medical Solutions, Erlangen, Germany). All imaging parameters used for the phantom and the lamb studies are given in Table 1. The repetition time (TR) was longer for the lamb study since a fat-saturation pre-pulse was used in this study, but not in the phantom study. *k*-space was evenly subsampled in the *ky*-phase encode direction, while the *kx*-read out and the *kz*-slice encode directions were both fully sampled in each dynamic time frame. All subsampled data were reconstructed with a previously described temporally constrained reconstruction (TCR) algorithm [53,54]. In the TCR algorithm, the series of temperature images are reconstructed by iteratively minimizing a cost function consisting of a temporal constraint term and a data fidelity term using a steepest descent method, according to

**Table 1:** MR scan parameters for the phantom and lamb studies, respectively

	**TR/TE (ms)**	**FOV (mm)**	**Voxel size (mm)**	**BW (Hz/px)**	**ETL**	**ES (ms)**	**FA (°)**	***t***_**acq **_**(s)**	***R***
**Phantom**	25/11	192 × 192 × 110	1.2 × 1.2 × 2.5	744	9	1.59	15	3.30	6
**Lamb**	36/11	192 × 144 × 60	1.0 × 1.0 × 2.0	752	9	1.61	20	4.32	4

*TR* repetition time, *TE* echo time, *FOV* field of view, *BW* bandwidth (in readout direction), *ETL* echo train length, *ES* echo spacing, *FA* flip angle, *t*_acq_ acquisition time, *R k*-space reduction/subsampling factor.

(1) $$m=\arg\;\min_{m\prime }\left({∥\mathrm{WFm}\prime -d'∥}_{2}^{2}+\alpha \sum_{i=1}^{N}{∥\nabla_{t}m\prime_{i}∥}_{2}^{2}\right)$$

where *m* are the artifact-free images, *W* is a binary matrix representing sampled phase encode lines, *F* is the fast Fourier transform, *m′* is the image estimate, *d′* is the acquired subsampled *k*-space data, *α* is a spatially varying weighting factor, the sum is over all *N* pixels in the data set, *∇*_*t*_ is the temporal gradient operator, and ∥ * ∥_2_ is the L_2_ norm. Following TCR reconstruction, all data were zero-filled interpolated to 0.5-mm in-plane resolution and 1.0-mm slice thickness to minimize partial volume effects. MR temperature measurements were performed with the PRF shift method [49], where the phase difference between two images is scaled according to

(2) $$\Delta \text{T}=\frac{\Delta \varphi }{\gamma \alpha B_{0}\mathrm{TE}}$$

where Δ*T* is the change in temperature (°C), Δ*φ* is the phase difference between the two images (radians), *γ* is the gyromagnetic ratio (Hz/T), *α* is a thermal coefficient (here assumed to be −0.01 ppm/°C), *B*_0_ is the main magnetic field strength (T), and *TE* is the echo time (s). All image reconstruction and post-processing was performed using MATLAB (R2013a, The MathWorks Inc., Natick, MA, USA).

**Figure 1:**
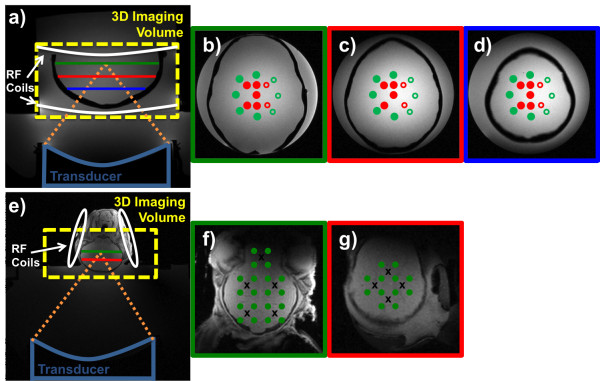
**Experimental setup. (a)** Coronal view of the experimental setup for the phantom study, where the yellow box indicates the 3D imaging volume and the white lines indicate the positions of the two 1-channel RF receive coils. Green, red, and blue lines indicate the positions of the three planes where the FUS was focused, and transverse views of these planes are shown in **(b–d)**. In the transverse views of the three focal planes, **(b–d)**, the solid red dots indicate the approximate mechanical focal positions, and solid green dots indicate the approximate electronically steered focal positions. Red and green rings indicate heatings “mirrored” from the left hemisphere to the right hemisphere. **(e)** Coronal view of the experimental setup for the lamb study, where the yellow box indicates the 3D imaging volume and the white lines indicate the positions of the two 2-channel RF receive coils. Green and red lines indicate the positions of the two planes where the FUS was focused, and transverse views of these planes are shown in **(f)** and **(g)**. In the transverse views in **(f)** and **(g)**, black crosses correspond to the transducer’s physical position, and the green dots correspond to the position of the electronically steered focal spot.

For the phantom study, two in-house-built radio-frequency (RF) receive-only single-channel loop coils were used for signal detection, and for the lamb study, two in-house-built dual-channel receive-only RF coils [55] were used (Figure 1a,e).

All FUS heating was performed with an MR-compatible phased-array ultrasound transducer (256 elements, 1-MHz frequency, 13-cm radius of curvature, focal spot full-width-at-half maximum 2 × 2 × 8 mm, Imasonic, Voray-sur-l’Ognon, France) and accompanying hardware and software for mechanical positioning and electronic beam steering (Image Guided Therapy, Pessac, France). The transducer was coupled to the phantom and the lamb skull with a bath of de-ionized and de-gassed water.

The phantom used in this study was composed of a single-layer polyvinyl chloride (PVC) plastic (speed of sound 2,330 m/s, specific heat capacity 1,047 J/kg*K, thermal conductivity 0.19 W/m*K [56,57]) skullcap (model A20, 3B Scientific, Tucker, GA, USA) embedded in tissue-mimicking gelatin (speed of sound 1,553 m/s, specific heat capacity 3,635 J/kg*K, thermal conductivity 0.55 W/m*K) [58]. A total of 29 sonications were performed in three planes, spaced 1.5 mm apart, utilizing both mechanical and electronic steering (Figure 1a–d). To minimize the number of sonications in this proof-of-concept study, it was assumed that the right and the left hemispheres were symmetric, and sonications were only performed in one hemisphere and then “mirrored” to the other hemisphere for treatment envelope evaluations. Each sonication was performed at 52 acoustic watts for 19.8 s, and after each heating, the phantom was allowed to cool for approximately 10 min.

For the lamb study, a recently euthanized lamb was placed in a supine position with the US focused through the skullcap (Figure 1e–g). The Institutional Animal Care and Use Committee approved all experiments. The lamb model was used since the thin skull enabled getting enough energy through the skull to cause substantial focal spot heating with our available FUS system. For this proof-of-concept study, no additional information would have been obtained if the animal had been alive, which is why the lamb was euthanized before the study. To ensure good acoustic coupling to the head and avoid trapping any air, the head was treated with hair removal cream before being placed on the FUS system. A total of 32 sonications were performed in two planes, spaced 8 mm apart. The transducer was mechanically moved to five positions in the plane more distal to the transducer and to four positions in the plane more proximal to the transducer, and from each mechanical position, the US was electronically steered ±5-mm in *x* and *y* (i.e., to the four “corners”). Each sonication was performed at 32 acoustic watts for 17.3 s, with 10 min of cooling in between sonications. No phase aberration correction of the US phases was applied in either the phantom or the lamb study.

Two different types of treatment envelopes were derived from the phantom and the lamb data. The first type utilized only the focal spot temperatures and demonstrates the type of envelope that can be derived from 2D MRTI with proper slice placement. In these envelopes, the hottest focal spot temperatures from the 29/32 heatings were extrapolated to the full intracranial volume. For the second type of envelope, the full 3D MRTI information was utilized, and the relative near-field heating was calculated as the ratio between the hottest focal spot voxel and the mean of the ten hottest near-field voxels inside the skull. Ten voxels were chosen as a compromise between reducing the standard deviation without introducing an underestimation of the temperature rise. For this second type of envelope, it is desirable that the ratio be as large as possible, i.e., for every degree of temperature rise occurring in the focal spot, only a fraction of that is occurring in the near field. These ratios were also extrapolated to the full intracranial volume.

Hydrophone scans (Model HNR-0500, ONDA, Sunnyvale, CA, USA) through the skullcaps of both the plastic skull (before it was embedded in the tissue-mimicking gelatin) and the lamb skull (post-treatment and with all soft tissue removed) were performed in a bath of de-ionized and de-gassed water to investigate the amount of beam aberration and intensity loss that were to be expected when focusing through the skulls. The hydrophone was attached to 2D stepper motors and a 20 × 20 mm FOV with a resolution of 0.25 × 0.25 mm was scanned both with and without the skullcaps in the beam path with a power of 2.7 acoustic watts.

## Results

**Figure 2:**
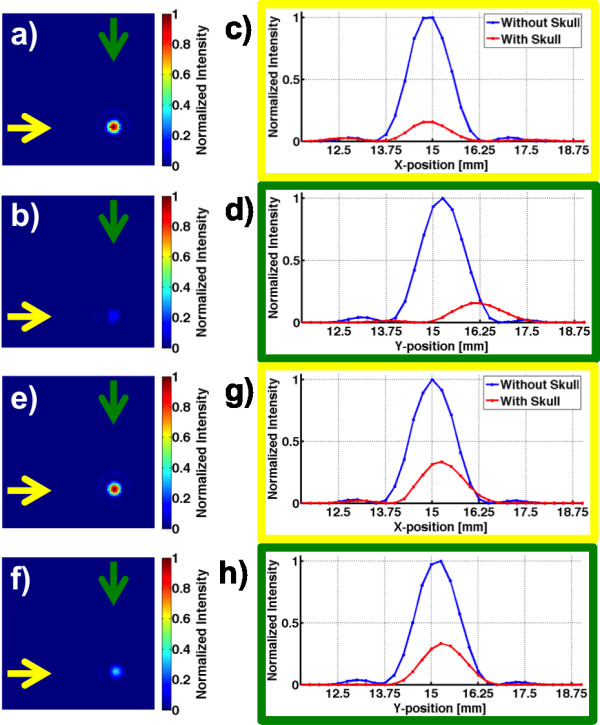
**Hydrophone scans result.** 2D plot of hydrophone scans without **(a)** and with **(b)** the plastic skull in the US beam path. Line plots in the *x*-direction (indicated by yellow arrows) and in the *y*-direction (green arrows) for the two cases are shown in **(c)** and **(d)**, respectively. All intensities are normalized to the maximum value in the water-only scan. The US focus is steered 5 mm in the *x*- and *y*-directions, relative to the geometric focus. Beam aberration is not significant; however, an intensity drop of about 85% and slight shift in the *y*-direction are seen to occur when focusing through the skull. **(e–h)** Corresponding data from the lamb skullcap after all soft tissue was removed. In this case, an intensity drop of about 70% and only slight beam aberration are observed.

**Figure 3:**
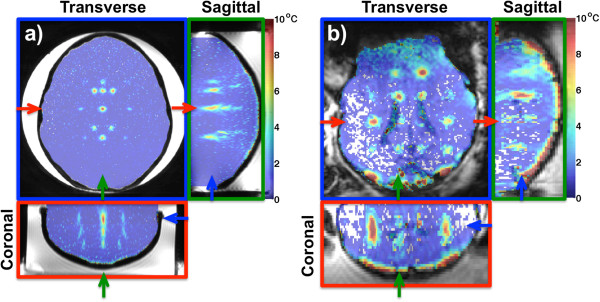
**Superposition of temperatures.** Three orthogonal views of superpositions of temperatures for the 29 sonications in the phantom study **(a)** and the 32 sonications in the lamb study **(b)**. In both **(a)** and **(b)**, the temperature maps are overlayed on magnitude images. Heatings in the right hemisphere in (a) are mirrored from the left hemisphere (see Figure 1). In both the phantom and the lamb studies, the focal spots are only slightly aberrated, in agreement with the hydrophone results in Figure 2. The near-field heating is more severe in the lamb study than in the phantom study. In **(a)** and **(b)**, the three views are color coded, with the colored frame and arrows indicating each view’s position in the other two views.

**Figure 4:**
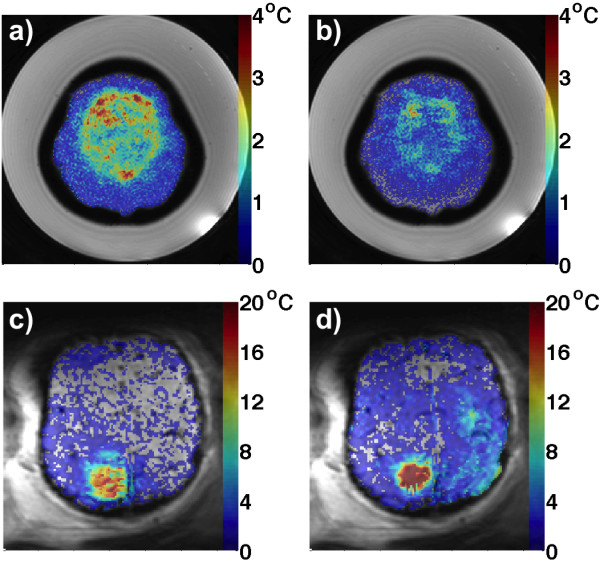
**Near-field MIPs.** Transverse 2D thin-slab MIPs of near-field temperatures (i.e., not including the focal spot) inside the skull for the phantom study **(a–b)** and the lamb study **(c–d)**. **(a)** and **(c)** show MIPs when focusing in the plane most distal to the transducer, and **(b)** and **(d)** show MIPs when focusing in planes more proximal to the transducer. In both cases, more severe near-field heating can be seen in the lamb study than in the phantom study, in agreement with Figure 3. All MPIs are overlayed on magnitude images.

**Figure 5:**
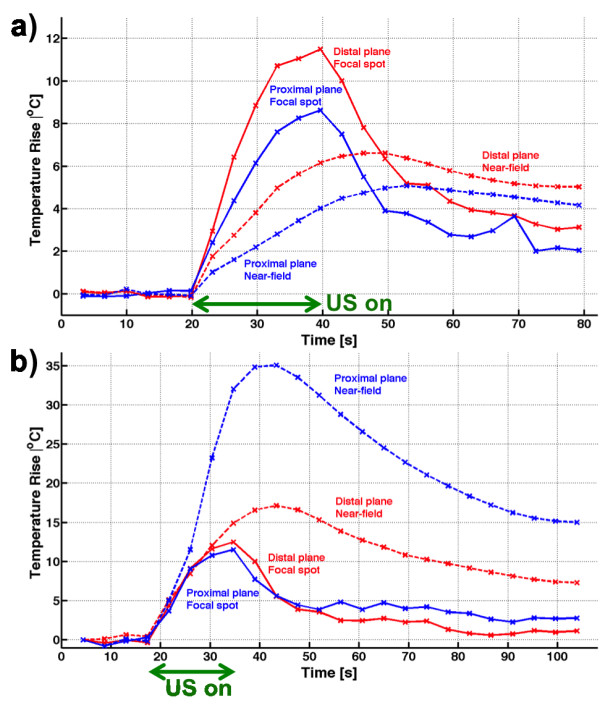
**Temperature rise vs. time.** Temperature rise as a function of time for **(a)** the phantom study and **(b)** the lamb study. Curves for both the hottest voxel in the focal spot (solid lines) and the mean of the ten hottest near-field voxels (dashed lines) are shown, both when focusing in the plane most distal to the transducer (red lines) and when focusing in a plane more proximal to the transducer (blue lines). For the phantom, the focal spot can be seen to heat more than the near field, whereas in the lamb study, the near field experiences greater temperature rises than at the focal spot. In both cases, the near field reaches the peak temperature after the US is turned off and cools slower than the focal spot. Green arrows indicate when the US was applied.

**Figure 6:**
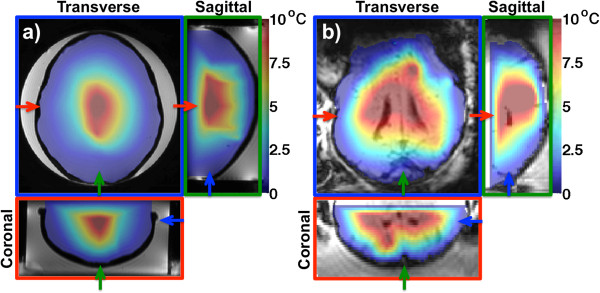
**Treatment envelopes based on 2D MRTI.** Three orthogonal views of the treatment envelopes based only on the focal spot heating for the phantom **(a)** and lamb **(b)** studies. Near-field temperatures are not considered for this type of envelope. Temperature rises of at least 10°C can be seen in large parts of the intracranial volume in both cases. This is the type of envelope that can be derived from 2D MRTI. In both **(a)** and **(b)**, the temperature maps are overlayed on magnitude images. In **(a)** and **(b)**, the three views are color coded, with the colored frame and arrows indicating each view’s position in the other two views.

**Figure 7:**
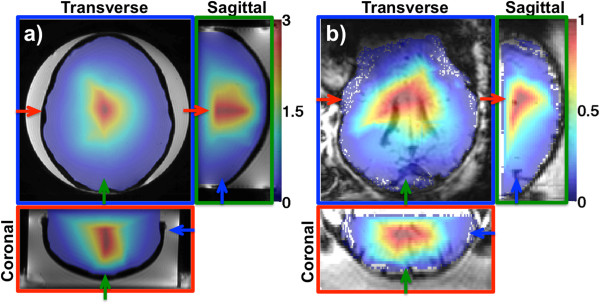
**Treatment envelopes based on 3D MRTI.** Three orthogonal views of treatment envelopes based on the relative near-field heating, calculated as the ratio between the hottest focal spot voxel and the mean of the ten hottest near-field voxels for the 29 and 32 heatings in the phantom **(a)** and lamb **(b)** studies. This type of envelope can only be derived from 3D MRTI data. In the phantom study, ratios of up to 3 can be observed for the most central regions. For the lamb study, the highest ratio observed is about 1, meaning that for the majority of the intracranial volume, the near-field region will be heated more than the focal spot. For both **(a)** and **(b)**, the temperature maps are overlayed on corresponding magnitude images. In **(a)** and **(b)**, the three views are color coded, with the colored frame and arrows indicating each view’s position in the other two views.

Figure 2 shows results from the hydrophone scans with and without the plastic and lamb skulls in the US beam path. In (a–b) and (e–f), 2D plots are shown for the plastic skull and lamb skull studies when electronically steering the US focus 5 mm in *x* and *y*. In (c–d) and (g–h), line plots through the focal spot in the *x*- and *y*-directions for the two studies are shown. All plots are normalized to the peak intensity measured in corresponding scans in the water bath only (i.e., without the skull in the US beam path). Intensity losses of about 85% (for the phantom study) and 70% (for the lamb study) were observed when focusing through the skulls. Only slight beam aberrations were observed in both cases, but in the phantom, a slight positional shift of the focus (~1 mm) in the anterior-posterior direction was observed.In Figure 3a,b, three orthogonal views of superpositions of the hottest time frame for all 29 and 32 sonications in the phantom and the lamb studies are shown. The focal spots can be seen to experience only slight beam aberration. The near field can further be seen to have more substantial temperature rise in the lamb study than in the phantom study. This is also highlighted in Figure 4, which shows transverse 2D thin-slab maximum intensity projections (MIP) of the tissue in the near field inside the skull (i.e., not including any focal spot heating, but just near-field voxels). The phantom, which can be placed closer to the transducer due to the larger acoustic window, experiences temperature rises in the near field of approximately 2°C–4°C, spread over a relatively large area (approximately 6 × 5 cm). For the lamb, the energy entering the skull is spread over a considerably smaller area (approximately 1 × 1.5 cm) because the transducer was located further from the skull, resulting in temperature rises of approximately 20°C–30°C.The temporal characteristics of the sonications are demonstrated in Figure 5 as the temperature rise as a function of time for the focal spot and the near field (mean of ten hottest voxels). Data for both the phantom and the lamb studies, and for both the most distal and the most proximal planes relative to the transducer, are shown.Figures 6 and 7 show the two types of treatment envelopes derived in this study. Figure 6 is based only on the focal spot temperatures, and Figure 7 is based on the relative near-field heating as the ratio between the focal spot and corresponding near-field heating, made possible by 3D MRTI. In Figure 6, temperature rises greater than 10°C can be seen in large parts of the brains for both the phantom and the lamb, suggesting that therapeutic levels of FUS can be delivered to these areas. Figure 7 shows that relative near-field heating ratios of up to 3 can be achieved in the phantom study, whereas for the lamb study, the majority of the brain has a ratio less than 1, indicating that the near field will experience more heating than the focal spot when attempting to treat these areas.

The reconstruction time for 250 iterations of the TCR algorithm for the phantom study was 803 s on a laptop with 2.5 GHz Intel Core i7 processor and 8 GB RAM (MacBook Pro, Apple Inc., Cupertino, CA, USA).

## Discussion

The methods presented in this study are transducer design independent, and the results highlight the need for treatment envelope studies to evaluate not only feasibility and efficacy, but safety as well. With 3D MRTI coupled with constrained reconstruction for improved spatial and temporal resolution, the fully insonified FOV can simultaneously be monitored and characterized. Substantially more information about possible unintended near-field heating, for improved safety, is available compared to envelope studies utilizing 2D MRTI.The hydrophone scans show that substantial loss in focal spot intensity occurs when focusing through both the plastic skull and the lamb skull. This is explained by the high US attenuation in the plastic and in the skull, and reflections occurring at the water/plastic or water/bone interfaces. It can be further noted that since the hydrophone scans are performed in the absence of phantom material and soft tissue, the intensity loss in actual experiments will be greater than the reported 85% and 70%, which is due only to the skulls. The focal spot shape does not experience any substantial amount of distortion in the hydrophone experiments presented, which supports not utilizing aberration correction for the FUS heating studies. The amount of focal distortion observed in both the phantom and the lamb is limited by the relative homogeneity of the skulls and the FUS setup used. CT scans (data not shown) show that the lamb skull is considerably more homogenous than a human skull (there is very little diploë in the lamb skull), so the variation in speed of sound for the individual beam paths through the skull is very limited. This is also the case for the plastic skull, which is composed of a single homogenous layer of PVC. For the lamb study, the transducer, which has a relatively small aperture, is placed far away from the skull. Hence, the beam paths from the individual elements all go through similar parts of the skull. The plastic skull was placed closer to the transducer than the lamb skull, but due to the limited size of the transducer, the difference in beam paths is still fairly small. From Figure 3a, it can be seen that all the focal spots in the phantom study are well defined. For the lamb study in Figure 3b, however, focal spots extending over the lateral ventricles can be seen to experience a corrupted focal spot shape due to the movement that the cerebrospinal fluid (CSF) in the ventricles experiences from the acoustic radiation force.

The sagittal and coronal views of Figure 3 also indicate that more severe near-field heating occurs in the lamb study than in the phantom study. This is further highlighted by the 2D MIPs of the near-field heating in Figure 4, showing that near-field heating of 2°C–4°C over a relatively large area in the phantom occurs, while in the lamb study, near-field heating of 20°C–30°C over a smaller area occurs. The main reason for the discrepancy is likely the fact that the larger size of the phantom allowed it to be positioned closer to the transducer (Figure 1a,e), and therefore, the incident US intensity was spread over a larger area at the plastic/gelatin interface than in the lamb study. The thermal properties of the plastic skull compared to the lamb skull can also have a limited effect. The specific heat of the PVC is higher than that of skull bone (1.05 kJ/kg*K vs. 0.44 kJ/kg*K), meaning that more energy is needed to heat the plastic skull; however, its thermal conductivity is lower (0.19 W/m*K vs. 0.32 W/m*K), meaning that it retains heat longer [56,57,59]. In the lamb study, the near-field heating is more severe when focusing more proximal to the transducer (Figure 4d), while this is not the case for the phantom study (Figure 4b). It can be hypothesized that the larger electronic steering of 1.5 cm between the different planes for the phantom study (compared to 0.8 cm for the lamb) results in greater intensity losses that counteract the fact that the power is spread over a smaller area.

The plots of temperature as a function of time in Figure 5 again show that more near-field heating occurs in the lamb study than in the phantom study. It can further be seen that the peak temperature of the near field occurs after the US is turned off and that the post-heating cooling is much slower than at the focal spot. These observations are in agreement with previously published results on tissue heating close to the bone [60,61] and are explained by the high US absorption and low specific heat and thermal conductivity of the bone. High US absorption and low specific heat result in the bone heating more easily than surrounding tissue, and the low thermal conductivity results in a delayed and prolonged heat delivery to the surrounding tissue.Treatment envelopes that are based only on the focal spot temperatures (Figure 6), which is what could be derived from 2D MRTI, suggest that with the US power applied, at least a 10°C temperature rise can be achieved in a large part of the intracranial volume. Of course, by increasing the power, even higher temperatures could have been achieved, and therapeutic levels of FUS could have been delivered to an even larger part of the intracranial volume. If these plots had been derived from actual 2D MRTI data, information about near-field heating would only be available at points where the 2D slices intercepted the skull. Taking into account the full 3D MRTI data by looking at the relative near-field heating in Figure 7, it can be seen that areas where Figure 6 suggests therapeutic levels of FUS can be delivered can in fact not be safely treated. This is especially true for the lamb study, where the ratio is less than 1 for the majority of the intracranial volume. This indicates that the temperature rise at the focal spot will be less than that at the skull. It should be noted that neither blood perfusion nor CSF circulation was present in the phantom or in the lamb study, and both would act as heat sinks both at the focal spot and in the near field in clinical studies. The presented treatment envelopes emphasize the need for envelope studies to incorporate not only feasibility and efficacy, but safety as well. It is shown that safety can be incorporated by monitoring the fully insonified 3D FOV and that this leads to a very different envelope than if only feasibility and efficacy are considered.

The amount of near-field heating observed in this proof-of-concept study is almost certainly more severe than what will be observed with the clinical tcMRgFUS systems used in previous treatment envelope studies [51,52] and current clinical trials [24]. These clinical systems operate at lower frequencies of 0.2 and 0.7 MHz that are more optimal for transcranial transmission and brain absorption [62,63] than the 1-MHz transducer used in this study. Compared to the transducer used in this study, the elements of the clinical transducers are also spread over a larger hemispherical surface that surrounds the head. This spreads the US energy over a larger surface area of the skull and thereby minimizes the near-field heating.

As in all iterative reconstruction methods, the reconstruction time of the TCR algorithm is fairly long. A real-time version of the TCR algorithm [64] has been presented and significantly decreases the reconstruction time to allow real-time availability of the reconstructed temperature maps. The reconstruction time is decreased by only applying the TCR algorithm over the focal spot region where the temperatures change most rapidly, and performs a significantly less computationally heavy sliding window reconstruction over areas where the temperature changes more gradually. The algorithm was implemented on a GPU to further speed up the reconstruction. However, for treatment envelope studies, real-time availability of the temperature maps is generally of secondary interest.

## Conclusions

This study demonstrates the concept and importance of assessing treatment envelopes in terms of both safety and efficacy. The treatment envelopes derived in this study incorporate safety assessments in terms of near-field heating evaluations and show that due to unintended near-field heating, it is not necessarily safe to treat in all areas where therapeutic levels of FUS can be delivered. The methods described in this study can be utilized to maximize the treatment envelope when designing new transducers for tcMRgFUS, as well as to evaluate existing systems. Since this proof-of-concept study was performed utilizing a non-optimal transducer design, and only in a phantom and a lamb model, more studies with clinical tcMRgFUS systems and human cadaver skulls will be necessary to derive meaningful treatment envelopes for these systems. The results in this study further indicate that from a safety point of view, tcMRgFUS treatments are ideally monitored with 3D MRTI covering the fully insonified field of view.

## Competing interests

The authors declare that they have no competing interests.

## Authors’ contributions

HO and JdB performed the MRgFUS experiments. HO conducted the data reconstruction and analysis, and drafted the manuscript. SA and DAC were responsible for the hydrophone equipment, and SA and HO performed the hydrophone experiments. HO, NT, AP, JWS, and DLP designed the study. HO, NT, AP, and DLP developed and implemented the software algorithms used. AF manufactured the skull phantom. All authors read and approved the final manuscript.
